# An Environmental Management Maturity Model of Construction Programs Using the AHP-Entropy Approach

**DOI:** 10.3390/ijerph15071317

**Published:** 2018-06-23

**Authors:** Libiao Bai, Hailing Wang, Ning Huang, Qiang Du, Youdan Huang

**Affiliations:** School of Economics and Management, Chang’an University, Middle Section of South Second Ring Road, Xi’an 710064, China; LB.Bai@chd.edu.cn (L.B.); hailing711@163.com (H.W.); Ning_Huang@chd.edu.cn (N.H.); Huangyoudan@chd.edu.cn (Y.H.)

**Keywords:** environmental management, management maturity model, construction program, AHP-entropy

## Abstract

The accelerating process of urbanization in China has led to considerable opportunities for the development of construction projects, however, environmental issues have become an important constraint on the implementation of these projects. To quantitatively describe the environmental management capabilities of such projects, this paper proposes a 2-dimensional Environmental Management Maturity Model of Construction Program (EMMMCP) based on an analysis of existing projects, group management theory and a management maturity model. In this model, a synergetic process was included to compensate for the lack of consideration of synergies in previous studies, and it was involved in the construction of the first dimension, i.e., the environmental management index system. The second dimension, i.e., the maturity level of environment management, was then constructed by redefining the hierarchical characteristics of construction program (CP) environmental management maturity. Additionally, a mathematical solution to this proposed model was derived via the Analytic Hierarchy Process (AHP)-entropy approach. To verify the effectiveness and feasibility of this proposed model, a computational experiment was conducted, and the results show that this approach could not only measure the individual levels of different processes, but also achieve the most important objective of providing a reference for stakeholders when making decisions on the environmental management of construction program, which reflects this model is reasonable for evaluating the level of environmental management maturity in CP. To our knowledge, this paper is the first study to evaluate the environmental management maturity levels of CP, which would fill the gap between project program management and environmental management and provide a reference for relevant management personnel to enhance their environmental management capabilities.

## 1. Introduction

Urbanization is the process of the population moving from rural areas to urban areas, and it represents an important economic development issue and leads to many economic, social and environmental changes [1]. By 2020, 60% of the Chinese population will live in cities and 45% will have an urban household registration [2]. To provide basic living features, such as entertainment and shopping, the Chinese government continuously encourages the expansion of construction projects to satisfy the demand for urban infrastructure development. This emerging development strategy has prompted the implementation of construction programs that play a crucial role in determining the urban infrastructure quality [3,4].

A construction program (CP) is a set of multiple interrelated projects [5], where each subproject of this set shares resources and cooperates with the other subprojects to achieve an efficient configuration and promote scientific decision-making [6]. To promote the implementation of CPs, many scholars have proposed construction models (CMs) to study these programs [7,8,9,10]. For example, Subiyakto and other scholars believed that CPs should be based on modern information technology; therefore, they recommended the scientific and dynamic arrangement of such programs using a modern construction program information platform [11]. Canbaz, Li and Bai et al. analyzed the interactive relationship between different construction projects and advocated that collaboration between projects should be promoted to guide and synergistically optimize the implementation of CPs [12,13,14]. Many other scholars have studied the dynamic optimization methods of CPs [15,16]. Wang and Shi believed that management elements, including the quality, schedule, cost, and risk, are the basis for the optimization of CPs, and they suggested that project managers carry out the program via the dynamic configuration of management elements [17,18]. As an alternative to protecting individual activities, Koo proposed a buffer allocation strategy by which periodic buffers are allocated in the flows of program constraint resources to stabilize a master construction schedule, and they illustrated the performance of the proposed strategy in terms of the program goals of productivity, flexibility, and long-term stability through comparative experiments using Monte Carlo simulations [19]. An et al. proposed a construction project management model with synergy optimization of multiple goals to study the orientation and synergy management of the whole process of the construction project, and they provided a reference for the synergy management and optimization of CPs [20]. At the spatial management level, Kazak and Tavana proposed the Spatial Decision Support System concept to support the selection of strategies for CPs [21,22]. Furthermore, many studies have also explored the role of managers and organizations in complex CPs [23,24,25]. Although many studies have been performed on CPs, little attention has been devoted to their environmental management capability [26].

The concept of environmental management was first proposed by the United Nations Environment Program and the United Nations Conference on Trade and Development (UNCTAD) in 1974, and it is defined as a method of coordinating the major development goal of meeting “all basic needs of humanity” and developing a plan to “meet basic needs of society but not beyond the biosphere’s allowable limits” [27]. According to this definition, environmental management capability is concerned with an organization’s ability to perform project activities in an environmentally friendly manner while attaining financial gains [28]. Organizations are generally considered responsible for responding to the environmental concerns of their operations [29]. This capability is often characterized by the adoption of an environmental management system standard [30], the evaluation of stakeholders’ environmental performance, and the development of an environmental policy to mitigate negative environmental impacts in organizations’ operations [28,29,30,31]. Therefore, the environmental management capability of a CP is defined as the ability to develop and adapt new environmental management practices as CPs are implemented to adapt to the requirements of environmental protection work in the future [32,33,34]. The development goal to “accelerate the establishment of a legal system and policy orientation for green production and consumption, establish and improve a green and low-carbon recycling economic system” was proposed in the 19th National Congress of the Communist Party of China (CPC) Congress, and environmental management issues have received unprecedented attention in China [35]. Harmonious development with the environment has also become a prerequisite for the implementation of CPs, which poses a challenge to the environmental management capabilities of these programs. Therefore, identifying and improving the environmental management capability of CPs while promoting harmonious development with the environment are practical issues that must be addressed in the urbanization process [36]. Some research shows that multinational contractors have been relatively proactive in environmental management. Chen and other scholars employed content analysis the extract and measure the degree of proactivity. They find that pollution abatement on-site is the important factor of impacts on firm short and long-term financial performances of multinational construction firms among the environmental practices [37]. Hengky et al. examined the effect of the combination of corporate environmental strategy and environmental uncertainty by environmental management accounting. They find the environmental management accounting is a useful and important tool for providing information to achieve superior corporate environmental performance in firms [38]. Park and Ahn analyzed strategic environmental management types within the Korean construction industry, evaluating the strategic stages of Korean construction industries based on Hunt and Auster’s model [39]. Ye and Yuan et al. developed a model for evaluating the environmental performance of construction waste management by using a system dynamics approach and how the dynamics interactions can influence the environmental performance of construction waste management [40]. Hossain compared the environmental performance of building construction waste management system in Hong Kong and provide guidelines to design an effective and resource-efficient building construction waste management system [41]. Based on the theory of planned behaviors, Ding, Yi et al. developed a system dynamic model of construction waste reduction management at the construction phase to simulate the environmental benefits of construction waste reduction management [42]. However, few researchers have studied the evaluation system and promotion path for the environmental management capabilities of CPs [26]. Therefore, the literature cannot provide guidance for organizations to improve their environmental management capabilities effectively.

In order to fill the gap of scant attention paid to the environmental management evaluation system, this paper proposes to creatively evaluate construction project environmental management capability and the environmental management maturity level of CPs. First, this paper reinstitutes dimensional of environmental management capability based on an in-depth study and analysis of the ten areas of project management from the Project Management Institute (PMI). Then, this paper employed the Analytic Hierarchy Process (AHP)-entropy method to assess the maturity level of the environmental management capabilities of CPs. This work also provides an examination of the effectiveness and feasibility of the proposed model by conducting a real computational experiment in Anhui Province, China. Thus, this study not only enriches the theories of CP management but also serves as a reference for the public and private sectors with regard to environmental management goals for sustainable development. The rest of this paper is organized as follows: Section 2 illustrates the research methodologies of this study. Section 3 describes a content analysis targeted at building the environmental management maturity model of CPs with the help of the AHP-entropy method and management maturity model. Section 4 presents a computational experiment in Anhui Province to verify the effectiveness and feasibility of this model. Section 5 discusses the results of this study. Finally, the last section presents the conclusions.

## 2. Methodologies

### 2.1. AHP-Entropy

Many approaches can be used to determine the weight of indexes in the existing research literature, and they are mainly divided into two categories: (1) subjective weight determination methods, which are represented by the Delphi method and AHP; and (2) objective weight determination methods, which are represented by the correlation coefficient method and the entropy method. Both of these approaches have certain deficiencies. The weights of the indexes determined by subjective methods can reflect researchers’ intentions but are often limited by the researchers’ knowledge and experience; thus, they exhibit a considerable level of arbitrariness and subjectivity. Moreover, the weights calculated using objective weight determination methods are closely linked with the actual data, although they are vulnerable to extreme values. To avoid these deficiencies and improve the accuracy of the weight values, the methodology selected and applied in this paper utilizes the AHP along with the entropy method. 

#### 2.1.1. Analytic Hierarchy Process

The AHP is a classic method for determining weights, and it is suitable for situations in which people’s qualitative judgments play a major role and the decision-making results cannot be measured directly and accurately. The AHP was proposed by Satty in 1971 to satisfy the immense difficulties of decision-making circumstances that involve multiple or even clashing criteria [43], and it is a multi-criteria decision-making method that combines qualitative and quantitative analyses by building a hierarchical model to describe the decision-making process in mathematical language. The underlying model provides a simple decision-making method for solving multi-objective and multi-criteria problems with unstructured characteristics. For this reason, the AHP is widely used in the field of evaluation. The use of the AHP for solving complex decision-making problems generally includes five steps [44,45,46,47,48]:

*Step 1*: Establish a hierarchy model. This involves breaking down the problem issue into various components and then structuring the components in hierarchical form.

*Step 2*: Construct the judgment matrix A. This step entails utilizing a 1–9 scale as in Table 1 to measure the results of pair-wise comparisons and setting priorities on every level of the hierarchy according to the organized structure given by the AHP.

*Step 3*: Calculate the judgment matrix A. In this step, the eigenvalues and eigenvectors that satisfy the Equation (1) are calculated:

(1) $$AW=\lambda_{max}W$$

where $\lambda_{max}$ is the largest eigenvalue and W is the normalized eigenvector corresponding to $\lambda_{max}$. The component i of vector W, denoted as $w_{i}$, is the weight of the corresponding element according to the sorting result.

*Step 4*: Produce a consistency check, which represents a critical step. To perform a consistency check for the decision matrix, the following steps are required:

First, calculate the consistency index (C.I.):

(2) $$C.I.=\frac{\lambda_{max}-n}{n-1}$$

where n is the matrix size.

Second, ascertain the random consistency index (R.I.) according to Table 2.

Third, calculate the consistency ratio (*C.R.*):

(3) $$C.R.=\frac{C.I.}{R.I.}$$

The C.R. is acceptable if its value does not exceed 0.10; otherwise, the judgment matrix is considered inconsistent. To obtain a consistent matrix, the judgments should be reviewed and improved.

*Step 5*: Calculate the weights, which is performed by completing Steps 1–4 for all levels in the hierarchy.

#### 2.1.2. Entropy

The entropy method is an objective weight determination method, and the evaluation results of index weights calculated with the entropy method are more reliable and accurate than those determined by subjective weighting methods. This method can be used to calculate the information entropy of the indexes. The weights for each index are subsequently determined according to the influence of the relative change of that index on the whole system, with larger weights assigned to indexes with a greater influence of relative change. If the entropy of an index is smaller, the variation degree of that index value is greater; therefore, the amount of information provided by that index is greater and accordingly receives a larger weight. Conversely, if the entropy of an index is large, then the variation degree of that index value is smaller; therefore, it provides less information and receives a smaller weight. Therefore, the key to determining the entropy weights is to construct the evaluation matrix X, and the structure of the option execution lattice is shown in Table 3. In Table 3, $C=\left\{C_{1},C_{2},C_{3\;},\cdots ,C_{n}\right\}$ is the set of evaluation indexes, n is the number of evaluation indexes, $B=\left\{B_{1},B_{2},B_{3},\cdots ,B_{m}\right\}$ is the set of evaluation objects, m is the number of evaluation objects, and $X_{mn}$ is the evaluation value of index $C_{n}$ for the object $B_{m}$.

According to the classic model of the entropy weight method [47,48] and following Wang and Wang [49,50], the entropy weight method based on the construction of the evaluation matrix can be performed according to the following steps:

*Step 1*: Standardize the evaluation matrix. In this step, a standardized appraisal matrix $X^{\prime }={\left(x_{ij}^{\prime }\right)}_{mn},\;i=1,2,\ldots ,m;j=1,2,\ldots ,n$ can be obtained by Equation (4):

(4) xij′={xij− i   min{xij} i   max{xij}− i   min{xij},Benefit indicator i   max{xij}−xij i   max{xij}− i   min{xij}, Cost indicator

where $x_{ij}^{\prime }$ represents the evaluation value of index $C_{j}$ for object $B_{i}$ after standardized and $x_{ij}^{\prime }\in \left[0,1\right]$.

*Step 2*: Calculate the specific density of the evaluation value of index $C_{j}$ for object $B_{i}$ and construct the specific density matrix Y:

(5) $$Y={\left(y_{ij}\right)}_{mn}$$

(6) $$y_{ij}=x_{ij}/\sum_{i=1}^{m}x_{ij}$$

*Step 3*: Calculate the entropy value of index $C_{j}$, denoted by $e_{j}$:

(7) $$e_{j}=-k\sum_{i=1}^{m}y_{ij}\;\ln y_{ij}$$

where k=1/lnm.

*Step 4*: Calculate the entropy weight of evaluation index $C_{j}$ as follows:

(8) $$w_{j}^{\left(E\right)}=\frac{1-e_{j}}{n-\sum_{j=1}^{n}e_{j}}$$

where $w_{j}^{\left(E\right)}$ is the weight of the evaluation index $C_{j}$ from the entropy method, and $1-e_{j}$ represents the information utility value of evaluation index $C_{j}$. Larger values for $1-e_{j}$ indicate the relative importance of evaluation index $C_{j}$ and imply larger values for the entropy weight of evaluation index $C_{j}$. Also, $0\leq w_{j}^{\left(E\right)}\leq 1$ and $\sum_{j=1}^{n}w_{j}^{\left(E\right)}=1.$ In essence, the entropy method takes advantage of the information utility value of each evaluation index in estimating the weights. A larger weight signifies greater importance given to its associated evaluation index. Therefore, the information utility value of evaluation indexes can be used and combined with the AHP to calculate their final weights.

#### 2.1.3. Combined Weighted Model Based on the AHP and Entropy

Parameter $w_{j}^{\left(A\right)}$ represents the weights of evaluation index $C_{j}$ obtained from the AHP method. Since the information utility value of an evaluation index reflects its importance, this value can be used to fine-tune $w_{j}^{\left(A\right)}$ according to Equation (9) [51]:

(9) $$w_{j}^{\left(F\right)}=w_{j}^{\left(A\right)}*\left(1-e_{j}\right),\;j=1,2,\ldots ,n$$

Then, the final weight of the evaluation index, denoted as $w_{j}$, can be obtained after the process of standardization:

(10) $$w_{j}=w_{j}^{\left(F\right)}/\sum_{j=1}^{n}w_{j}^{\left(F\right)}$$

### 2.2. Management Maturity Model

**Figure 1:**
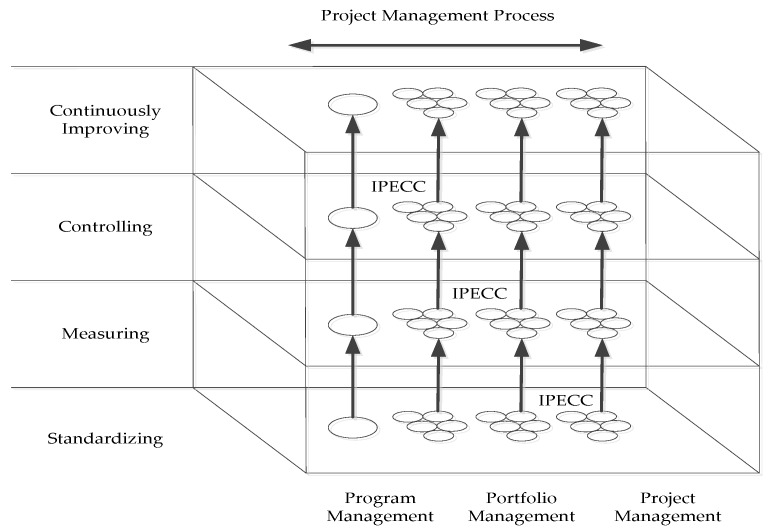
Framework of the OPM3.

The theory of maturity was developed for software quality evaluation to evaluate the capability of software contractors and help software companies improve the maturity of process quality [52]. Since then, this theory has been used to evaluate the level of project management capability and attracted widespread attention from experts in recent years. More than 30 different types of project management maturity models have been proposed. Among them, the most well-recognized model is the Organization Project Management Maturity Model (OPM3) proposed by the Project Management Institute (PMI) [53], which presents advantages in the areas of evaluation systems, model frameworks and applications. The OPM3 was developed and established based on the results of extensive, in-depth and effective testing implemented in a number of representative organizations; therefore, the results possess a certain level of practical feasibility [54]. According to the PMI, the OPM3 can be divided into three dimensions as shown in Figure 1.

The first dimension consists of the gradients of maturity, which are divided into four levels: standardizing, measuring, controlling and continuously improving. The second dimension is the process of project management, which consists of five steps: initiating process, planning process, executing process, controlling process and closing process (IPECC). The OPM3 is distinguished from other management maturity models by its third dimension. In this dimension, the scope of project management has been expanded from a single project to a project portfolio and program, which increases the concept of project management from a tactical level to a strategic one. Therefore, the OPM3 is recognized as the best-practice standard for assessing and developing capabilities in executing strategies through projects, and it provides a method for organizations to understand their organizational project management processes and practices and ensure that these processes are capable of performing successfully, consistently, and predictably. Additionally, organizations can position their project management capability in these three dimensions and accurately judge their maturity level through OPM3, which can help them develop a roadmap to improving performance.

## 3. Environmental Management Maturity Model of Construction Program

### 3.1. Environmental Management Index System of Construction Program

A CP is a set of many different construction projects, and the implementation of a CP substantially interferes with the ecological environment. Compared with those of a single construction project, the environmental impacts of CPs are characterized by their grouped pattern, systemic nature, cumulativeness, sweeping influence and potentiality. The impact of the implementation of a single construction project on the environment may be relatively small. However, the implementation of a CP tends to create interactive synergies between multiple projects, and the cumulative effect is relatively high. Therefore, the environmental management of CPs should focus on the synergies among different projects and take them into consideration when building the environmental management index system of the CP. Thus, the index system of a CP consists of two major components: the index system for a single project and the synergies of the environmental management indexes among different projects. According to the current construction procedures of Chinese infrastructure projects [54], the environmental management index system for a single project can be built based on the project’s life cycle and divided into five stages: initiating, planning, executing, controlling and closing. Therefore, the environmental management index system of CPs can be constructed according to the existing literature [26,55,56,57,58,59], as shown in Table 4.

### 3.2. Dimensions of the Environmental Management Maturity Model of CP

**Figure 2:**
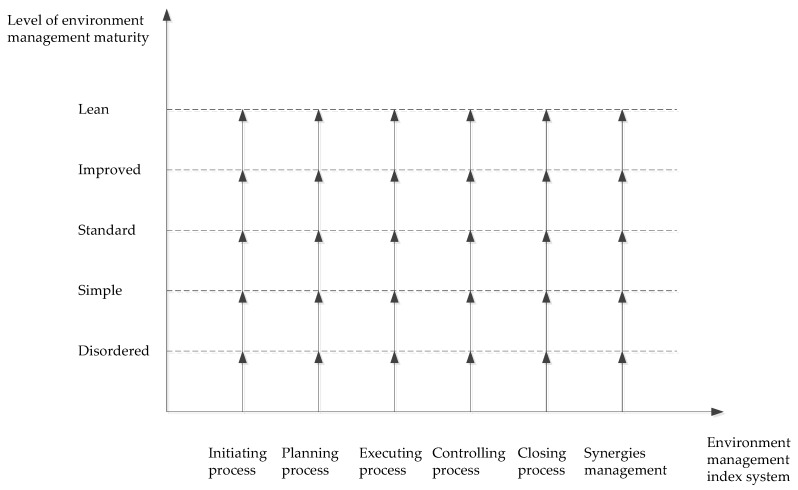
Dimensions of the Environmental Management Maturity Model of Construction Program (EMMMCP).

According to the OPM3, the model should have three dimensions. However, in this paper, since the focus of our research is CP, the scope of project management is fixed according to the objective of the program. Therefore, the three dimensions of the environmental management Maturity Model of CP (EMMMCP) can be simplified into a two-dimensional model that includes the environmental management index system and the level of environmental management maturity as the dimensions. Then, as shown in Figure 2, the dimensions of the EMMMCP can be obtained.

In Figure 2, the abscissa is the environmental management index system and reflecting the environmental evaluation indicator of the CP at different stages. The y-axis is the maturity level of environmental management, and it measures the degree of management orders and reflects the gradients of maturity. To increase the accuracy of the proposed system at determining the maturity level of Chinese organizations’ project management processes and provide an effective approach to improving their project management abilities, the development of national project management abilities must be considered when dividing the competency levels. Since project management in China has not yet reached the standards of European and American countries, the maturity levels of the current OPM3 model are less relevant; therefore, the maturity level classifications need to be refined. Therefore, when setting the maturity levels, the current levels of project management maturity must be subdivided to ensure the accuracy of competency positioning. In this paper, we divide environmental management maturity into the following five levels according to the principles proposed by Kerzner [60]: disordered, simple, standard, improved and lean. Combining the implementation characteristics of the CP, the maturity levels of environmental management can be obtained as shown in Table 5.

### 3.3. Assessment of the Environmental Management Maturity of a CP

#### 3.3.1. Calculation of the Importance of Decision-Makers

Assuming the criteria layer of the environmental management index system of the CP is the set IC={Initiating ,Planning ,Executing,Controlling,Closing,Synergy} = $\left\{IC_{1},IC_{2},IC_{3},IC_{4},IC_{5},IC_{6}\right\}$, then the index layer of the system can be denoted as $IC_{u}=\left\{IC_{u1},\;IC_{u2\;},\ldots ,IC_{uv}\right\}$, where u is the number of criteria layers, v is the number of index layers, and $IC_{uv}$ is the vth evaluation index of criteria layer $IC_{u}$. According to the preference extraction scheme of group decision theory [61,62], assume that the set of decision-makers is given by $DM=\left\{DM_{1},DM_{2},\ldots ,DM_{z}\right\}$ and each decision-maker scores the importance of all other decision-makers in the criteria dimension and assigns the importance score z,z−1,z−2,…,1 to each decision-maker in order from the important to the unimportant. Then, the importance score set of each decision-maker to others can be obtained:

(11) $${\mathrm{IM}}^{s}=\left[\begin{array}{cccc} im_{11}^{s} & im_{12}^{s} & \cdots & im_{1a}^{s}\\ im_{21}^{s} & im_{22}^{s} & \cdots & im_{2a}^{s}\\ \cdots & \cdots & \cdots & \cdots \\ im_{z1}^{s} & im_{z2}^{s} & \cdots & im_{za}^{s} \end{array}\right]$$

In this equation, s=(1,2,…,z), $im_{sk}^{s}$ is the importance score set of the decision-maker of the criteria dimension k with respect to himself, such that $im_{sk}^{s}=z$. Organizing these sets, the importance matrix of all decision-makers could be obtained:

(12) $$IM=\left\{im_{sk}\right\}=\left[\sum_{i=1}^{z}im_{sk}\right],k=1,\;2,3,\ldots ,\;a.$$

Normalizing the matrix IM, the decision-maker status matrix $IM^{\prime }$ could be obtained:

(13) $$im_{sk}^{\prime }=\frac{im_{xk}}{\sum_{x=1}^{z}im_{xk}}$$

Here, $im_{sk}^{\prime }$ is the relative importance of decision-maker s (s=1,2,…,z) to the criteria dimension k(k=1, 2,3,…, a), $im_{xk}$ is the importance score of the criteria dimension k, which is valued by decision-maker x, and $\sum_{x=1}^{z}im_{xk}$ is the algebraic sums of all decision-makers for the criteria dimension k.

#### 3.3.2. Determination of Maturity Level

The environmental management maturity level of the criteria and index layers can be scored by different decision-makers according to the scoring principles shown in Table 5, which are denoted as $SC=\left\{sc_{sk}\right\}$*.* Then, the comprehensive score of environmental management maturity can be obtained:

(14) $$Q_{k}=im_{sk}^{\prime }\cdot sc_{sk}\cdot w_{k}{}^{T}$$

where $Q_{k}$ is the comprehensive score of environmental management maturity, which indicates the environmental management maturity level of evaluation object k, and $w_{k}$ is the final weight of evaluation object k obtained via Equations (1)–(10) based on the methodologies of AHP and entropy. Then, the assessment results for the environmental management maturity of a CP can be calculated:

(15) $$Q=\sum_{k=1}^{m}Q_{k}.W_{k}$$

## 4. Computational Experiment and Results

PaFeng Township is a village populated by the Hui and Manchu people and located in the east of Hefei in Anhui Province. In 2010, this township started to promote urbanization and relocated the 10 communities within its jurisdiction. This program involved a large number of infrastructure construction projects, including the creation of residential buildings, transportation facilities, public common areas, entertainment and consumption facilities, and other projects. Consequently, the program is a relevant application of CP, which emphasizes the synergistic relationship between different projects. Furthermore, PaFeng Township is a small village with many ethnic groups, including the Hui, Manchu and Han people, whose environmental requirements, especially with regard to the living environment and natural environment, are relatively high. Therefore, determining the level of environmental management maturity of the CP and proposing improvement measures to meet environmental requirements are of important practical significance for the sustainable development of these ethnic groups. Thus, the CP of PaFeng Township has been chosen as an example for computational experiments in this paper to demonstrate the application and effectiveness of the EMMMCP.

### 4.1. Determination of the Initial Weight by AHP

In this paper, five decision-makers who passed Level A in the 4-L-C system proposed by the International Project Management Association (IPMA) were selected from the expert library of the construction project management committee (CPMC) of China’s construction industry association to assess the environmental management maturity of the CP. All of them are IPMP level A certificate holders and named Certified Project Directors according to IPMA, they are responsible for the management of a complex portfolio of an organization and the construction management of an important construction programme within an organization. This paper selected them as evaluators as they have proved their mastery of the system of modern construction project management knowledge and concepts and possess the project management ability for construction projects, or the power and qualification for managing construction projects contained in a programme. Therefore, the evaluation results of these experts with a certain scientificity and accuracy. According to Table 1 and Equation (11), the judgment matrix of the criteria layer, shown in Appendix A and importance score set of each decision-maker towards others $IM^{x}$, shown in Appendix B, can be obtained.

Analyzing the judgment matrix of the criteria layer, the initial weight values of the indexes for the criteria layer can be calculated using Equations (1)–(3). The values are shown in Table 6.

According to Table 6, the weights of the criteria layer obtained by the AHP are $W_{0}^{\left(A\right)}=\left[0.0349\;0.0532\;0.0825\;0.1443\;0.1698\;0.5154\right]$. Then, the initial weights of the index layer can be also obtained:

$W_{1}^{\left(A\right)}=\left[0.038\;0.073\;0.140\;0.259\;0.490\right]$
$W_{2}^{\left(A\right)}=\left[0.043\;0.076\;0.160\;0.219\;0.502\right]$
$W_{3}^{\left(A\right)}=\left[0.031\;0.035\;0.073\;0.084\;0.145\;0.247\;0.386\right]$
$W_{4}^{\left(A\right)}=\left[0.031\;0.077\;0.108\;0.174\;0.221\;0.390\right]$
$W_{5}^{\left(A\right)}=\left[0.027\;0.054\;0.073\;0.095\;0.164\;0.288\;0.300\right]$
$W_{6}^{\left(A\right)}=\left[0.028\;0.072\;0.095\;0.127\;0.265\;0.413\right]$

where $W_{0}^{\left(A\right)}$ is the criteria layer’s weight, and $W_{1}^{\left(A\right)}$, $W_{2}^{\left(A\right)}$, $W_{3}^{\left(A\right)}$, $W_{4}^{\left(A\right)}$, $W_{5}^{\left(A\right)}$ and $W_{6}^{\left(A\right)}$ are the index layer’s weights.

### 4.2. Final Weight Determination by the Combined Weight Model

Although the CP of PaFeng Township involves a large number of infrastructure projects, the main and most important construction projects are residential buildings $CP_{1}$, transportation facilities $CP_{2}$, square constructions $CP_{3}$, and entertainment and consumption facilities $CP_{4}$. Therefore, this paper has chosen these projects as the assessment objects and invited experts to rate their status levels, as shown in Appendix C. According to Appendix C, four types of CP have been selected for assessment; thus, *m* = 4. Then, the entropy weight of indexes can be obtained via Equations (4)–(8):

$W_{0}^{\left(E\right)}=\left[0.0529\;0.0981\;0.0711\;0.6548\;0.1080\;0.0151\right]$
$W_{1}^{\left(E\right)}=\left[0.3126\;0.0283\;0.0637\;0.4405\;0.1550\right]$
$W_{2}^{\left(E\right)}=\left[0.1423\;0.0155\;0.1307\;0.5322\;0.1792\right]$
$W_{3}^{\left(E\right)}=\left[0.0568\;0.0083\;0.0678\;0.5275\;0.0112\;0.0287\;0.2997\right]$
$W_{4}^{\left(E\right)}=\left[0.0147\;0.0552\;0.0694\;0.0393\;0.7209\;0.1005\right]$
$W_{5}^{\left(E\right)}=\left[0.0486\;0.3186\;0.1436\;0.1456\;0.2070\;0.1030\;0.0336\right]$
$W_{6}^{\left(E\right)}=\left[0.1696\;0.0451\;0.1188\;0.2085\;0.2059\;0.2520\right]$

where $W_{0}^{\left(E\right)}$ is the weight of the CP’s criteria layer obtained from the entropy method, and $W_{1}^{\left(E\right)}$, $W_{2}^{\left(E\right)}$, $W_{3}^{\left(E\right)}$, $W_{4}^{\left(E\right)}$, $W_{5}^{\left(E\right)}$ and $W_{6}^{\left(E\right)}$ are the entropy values of the CP’s index layer obtained from the entropy method. According to Equations (9) and (10), the final weights of these indexes can be obtained:

$W_{0}=\left[0.0138\;0.0390\;0.0439\;0.7076\;0.1373\;0.0583\right]$
$W_{1}=\left[0.0558\;0.0097\;0.0417\;0.5363\;0.3565\right]$
$W_{2}=\left[0.0261\;0.0050\;0.0894\;0.4960\;0.3835\right]$
$W_{3}=\left[0.0100\;0.0016\;0.0281\;0.2529\;0.0092\;0.0403\;0.6578\right]$
$W_{4}=\left[0.0021\;0.0195\;0.0343\;0.0313\;0.7326\;0.1801\right]$
$W_{5}=\left[0.0112\;0.1467\;0.0896\;0.1185\;0.2924\;0.2551\;0.0866\right]$
$W_{6}=\left[0.0231\;0.0158\;0.0551\;0.1296\;0.2670\;0.5093\right]$

where $W_{0}$ is the final weight of the CP’s criteria layer obtained by AHP-entropy, and $W_{1}$, $W_{2}$, $W_{3}$, $W_{4}$ $W_{5}$ and $W_{6}$ are the final weights of the CP’s index layer obtained by the AHP-entropy method.

### 4.3. Determination of EMMMCP Levels

The CP of PaFeng has been implemented for eight years; therefore, the level of environmental management maturity of these programs must be determined to propose improvement measures. To this end, these five decision-makers were also asked to assess the level of environmental management maturity of the CP of PaFeng Township based on the principles shown in Table 5 (the results are shown in Appendix D). Analyzing the importance score sets shown in Appendix B, the decision-maker status matrix $IM^{\prime }$ can be obtained via Equations (12) and (13):

$$IM_{0}^{\prime }=[\begin{array}{cccccc} 0.2062 & 0.2151 & 0.2100 & 0.1954 & 0.2045 & 0.2247\\ 0.1959 & 0.2366 & 0.1800 & 0.2069 & 0.2045 & 0.2472\\ 0.2268 & 0.1935 & 0.1800 & 0.1609 & 0.1818 & 0.1685\\ 0.1856 & 0.1398 & 0.2200 & 0.2299 & 0.1932 & 0.1685\\ 0.1856 & 0.2151 & 0.2100 & 0.2069 & 0.2159 & 0.1910 \end{array}]$$

Then, the level of PaFeng’s EMMMCP for the criteria layer indexes can be obtained via Equation (14):

$$Q^{\prime }=\left[\begin{array}{cccc} Q_{1}, & Q_{2}, & \ldots & Q_{6} \end{array}\right]=\left[2.0736,\;3.3138,\;3.4628,\;2.6119,\;3.8063,\;1.6279\right].$$

where $Q_{1},\;Q_{2},\;Q_{3},\;Q_{4},\;Q_{5},\;\mathrm{and}\;Q_{6}$ are the EMMMCP levels for the criteria layer indexes, which consist of the initiating process $I_{1}$, planning process $I_{2}$, executing process $I_{3}$, controlling process $I_{4}$, closing process $I_{5}$ and synergy process $I_{6}$, respectively. Therefore, the overall level of PaFeng’s EMMMCP can be calculated according to Equation (14):

$$Q=\sum_{k=1}^{m}Q_{k}\cdot W_{k}=Q^{\prime }\cdot W_{0}{}^{T}=2.7759.$$

where Q is the overall level of PaFeng’s EMMMCP, $Q^{\prime }$ is the level of PaFeng’s EMMMCP for the criteria layer indexes, $W_{0}$ is the final weight of the CP’s criteria layer indexes obtained by the AHP-entropy method. These results show that the level of PaFeng’s EMMMCP from highest to lowest are: the closing process $I_{5}$, executing process $I_{3}$, planning process $I_{2}$, controlling process $I_{4}$, initiating process $I_{1}$ and synergy process $I_{6}$. And, the initiating process $I_{1}$ and synergy process $I_{6}$ are the lowest. Therefore, if managers want to improve the level of PaFeng’s EMMMCP effectively, $I_{6}$ and $I_{1}$ are the key processes to be addressed and emphasized first.

## 5. Discussion

According to the Figure 2, Table 5 and the calculation results of PaFeng’s EMMMCP levels, the conclusion could be obtained that the maturity level of environmental management for CP overall for this township is standard and not ideal. Therefore, the theoretical standards of environmental management for PaFeng’s CP have been constructed, but the synergistic effects on the environment have not been analyzed from the whole perspective.

In addition, the maturity level of environmental management for the different processes of PaFeng’s CP, including initiating, planning, executing, controlling, closing and synergies, are designated standard, improved, improved, standard, improved and simple, respectively. The reason for this assessment is that the construction form of PaFeng’s CP follows a classic model of CP management that emphasizes the synergistic relationship between different projects. However, the implementation of this program follows a traditional model that decomposes these programs into individual projects, and many subcontractors pay attention only to their assigned tasks and short-term interests within their own contracted project while neglecting the goals of the overall program for management of the environment, which results in a lack of coordination between the environmental management capabilities of different projects. Consequently, the environmental management system of PaFeng’s CP is insufficient to ensure the continuous improvement of environmental management capability.

**Figure 3:**
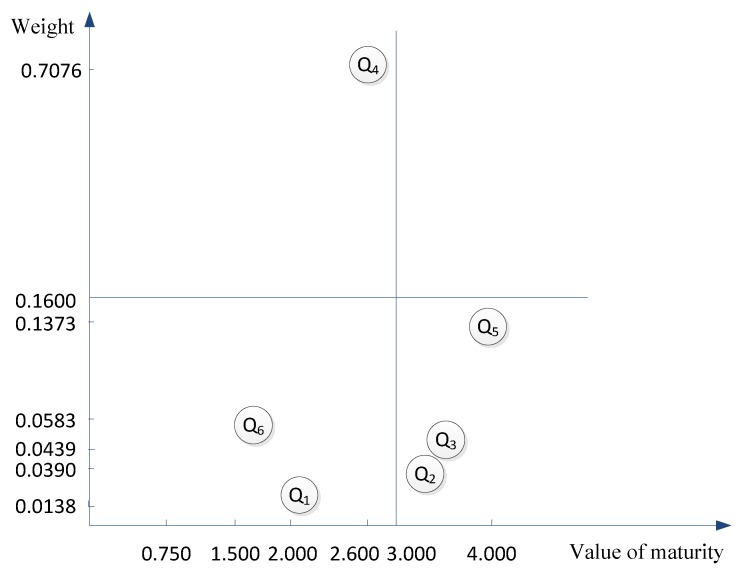
Four quadrants of PaFeng’s Environmental Management Maturity Model of Construction Program (EMMMCP).

Moreover, the results indicate that although the initiating and controlling processes are at the same level overall, their individual levels of maturity and the weights between them are very different. Thus, further analysis of the results is necessary to understand how to improve the level of environmental management maturity. Therefore, the maturity level and index weights are selected as the dimensions of analysis in this paper. Averaging the weights of these 6 indexes yields 0.16 as the critical value for dividing the ordinate quadrant, and an average maturity level of 3 as the critical value for dividing the abscissa quadrant. Then, the results can be displayed through a four-quadrant approach as shown in Figure 3.

In Figure 3, the controlling process $I_{4}$ is in the 2nd quadrant, where the environmental management maturity level is low, but the value of the weight is large, meaning that the controlling process $I_{4}$ has a significant impact on the level of environmental management maturity, but the management results are very poor. Therefore, $I_{4}$ is also a key process to be addressed and exhibits substantial room for improvement. Furthermore, the initiating process $I_{1}$ and synergy process $I_{6}$ are in the 3rd quadrant, which indicates that $I_{1}$ and $I_{6}$ are also key processes that should be emphasized first. Moreover, none of the implementation processes of PaFeng’s CP are located in the first quadrant, which means that the environmental management maturity level is very low and needs to be enhanced further. The planning process $I_{2}$, executing process $I_{3}$, and closing process $I_{5}$ are in the 4th quadrant, which means that their proportion is small, but their management effects are very good. For this reason, managers should not emphasize $I_{2}$, $I_{3}$ and $I_{5}$ and should instead focus on the implementation processes located in other quadrants. Therefore, the processes of $I_{1}$, $I_{4}$ and $I_{6}$ should be emphasized if the managers want to improve the environmental management maturity level of PaFeng’s CP.

## 6. Conclusions

The accelerating process of urbanization in China has led to good opportunities for the development of construction projects, although environmental issues will gradually become an important constraint on these projects. Many projects have shown that effective environmental management must be conducted to reduce the negative impact on the environment caused by CPs. Environmental protection factors should also be incorporated into all implemented processes of CPs. Simultaneously, the cumulative environmental impact of cascade development projects should be considered to maintain ecological balance and the coordinated development of the economy, society, and the natural environment.

To describe the environmental management capability of projects quantitatively, this paper proposed a 2-dimensional EMMMCP based on an analysis of existing theories of project group management and management maturity models. In this model, synergetic processes were considered while building the 1st dimension, which consisted of the environmental management index system to compensate for the lack of consideration of synergies in previous studies. The maturity level of environmental management was then taken as the 2nd dimension and constructed by redefining the hierarchical characteristics of CP environmental management maturity levels to include a “disordered level”, “simple level”, “standard level”, “improved level” and “lean level”. Then, mathematical descriptions and a solution to this proposed environmental management Maturity Model were provided using the AHP-entropy method. To verify the effectiveness and feasibility of this proposed model, a computational experiment was conducted. The results suggest that this model is reasonable for evaluating the level of CP environmental management maturity levels and could provide a reference for relevant management personnel to enhance their environmental management capabilities from the following aspects:

(1) Pay more attention to the environmental management of synergy process: according to the results of model analysis, environmental management maturity level of synergy process is relatively low which is consistent with the reality. The reasons of the phenomenon are the implementation phases are relatively clear, the phase transition content more specifically and environmental management is relatively simple for a single project, but the division boundary of CPs’ implementation phases is fuzzy, the content of synergetic management is more complex and the environmental management standards among different projects are not uniform. Therefore, to improve the CP environmental management maturity level, organizations should develop the implementation standards from the perspective of CPs, and forming a standardization, standardization and scientization mechanism of CPs, to ensure the effective transition and scientific synergy between different implementation processes of CPs.
(2) Environmental management ability training objectives should be fixed in the initiating process: nowadays, the Chinese Government has increasingly emphasized the construction enterprise environment management capacity and the adaptability to environment. Initiating process is the starting point for the implementation of the CP, setting up the promotion objective of environment management ability has an important guiding role to promote enterprise sustainable development, which could ensure the CP to carry and realize the goal of environmental management. Therefore, enterprises should set clear objectives for environmental management capabilities in the initiating process, develop a strict assessment of rewards and punishment mechanism and raise environment management unification consciousness in order to enhance the competitiveness within the increasingly important context of environmental protection.
(3) Accumulating the environmental management experience of CPs in the controlling process. The objective of promoting the environmental management capabilities is realized in the controlling process of a concrete CP. However, China’s engineering project companies started to pay attention to environmental management is relatively late, the environmental management experience of the construction project, especially the environmental management experience of CPs, is still relatively weak, which is unable to provide theoretical support for the management of CP companies. Therefore, enterprises should establish an environmental management knowledge library of CPs by collecting, sorting, analyzing and summarizing the typical cases of CPs’ environmental management, and put forward countermeasures and suggestions for different types of CPs’ environmental management, providing a reference for decision makers to set environmental management strategies for improving their environmental management capacity.

In the PMBOK, PMI defined the overall framework of project management and proposed ten areas of project management including project scope management, project time management, project cost management, project quality management, project human resources management, project communications management, project risk management, project procurement management, project integration management, project stakeholders management, however, an in-depth study and analysis of the area of the project environmental management is lacking. With the increasing emphasis on environmental issues throughout the world, the realization of harmony between construction projects and the environment will inevitably become an important prerequisite for the implementation of constructions in the future. Therefore, the research on how to improve the environmental management capability of engineering construction enterprises proposed in this paper can provide a theoretical support for the sustainable development of enterprises, enrich and improve the discipline theory of project management and provide some supports for the development of discipline project management. To our knowledge, this paper is the first study to evaluate the environmental management capability and the environmental management maturity level of CPs, which not only enriches the theories of CP management but also serves as a reference for the public and private sectors with regard to environmental management goals for sustainable development. However, this paper is also subject to certain limitations. One limitation is that only 5 experts were selected to ensure the quality of the article. In addition, the development of environmental management maturity in China is still in an initial stage. Some of the benefits in the area of CPs have not yet been realized, such as cost reductions and health performance improvements. What is more, the situation of these CPs, especially ongoing a planned CPs at the area have not been presented, which could not support the readers to understand the spatial characters of them. These limitations should be studied and developed in the future.

## Figures and Tables

**Table 1:** Scale from 1 to 9 of absolute number.

Intensity of Ce	Level of Importance
1	Same importance
3	Moderate importance
5	Strong importance
7	Too strong importance
9	Extremely strong importance
2,4,6,8	Middle values

**Table 2:** Values of the random consistency index *R.I.*

N	1	2	3	4	5	6	7	8	9
*R.I.*	0	0	0.58	0.90	1.12	1.24	1.32	1.41	1.45

**Table 3:** Structure of the evaluation matrix.

Index Object	$C_{1}$	$C_{2}$	$C_{3}$	……	$C_{n}$
$B_{1}$	$x_{11}$	$x_{12}$	$x_{13}$	……	$x_{1n}$
$B_{2}$	$x_{21}$	$x_{22}$	$x_{23}$	……	$x_{2n}$
……	……	……	……	……	……
$B_{m}$	$x_{m1}$	$x_{m2}$	$x_{m3}$	……	$x_{mn}$

**Table 4:** Environmental management index system of CPs.

Target Layer	Criteria Layer	Index Layer
Environmental management index system of CP	Initiating process $I_{1}$	Adequacy of project data collection $I_{11}$
		Capability of project needs analysis $I_{12}$
		Level of the project feasibility study $I_{13}$
		Capability of project planning $I_{14}$
		Analysis of the accuracy of the project’s surrounding environment $I_{15}$
	Planning process $I_{2}$	Planning and treatment of the impact on the ecological environment $I_{21}$
		Capability of project planning $I_{22}$
		Capability of investment planning $I_{23}$
		Capability of quality planning $I_{24}$
		Capability of risk planning $I_{25}$
	Executing process $I_{3}$	Impact on the ecological environment $I_{31}$
		Efficiency of the project team $I_{32}$
		Capability of project execution $I_{33}$
		Capability of project tracking $I_{34}$
		Capability of project contract management $I_{35}$
		Capability of project information management $I_{36}$
		Capability of handling project conflict $I_{37}$
	Controlling process $I_{4}$	Capability for controlling the negative impact on the environment $I_{41}$
		Ability to control the project schedule $I_{42}$
		Ability to control the project cost $I_{43}$
		Ability to control the project quality $I_{44}$
		Ability to control the risk identification $I_{45}$
		Ability to control the sudden changes in the project $I_{46}$
		Capability of eco-environmental impact assessment $I_{51}$
	Closing process $I_{5}$	Project completion rate on schedule $I_{52}$
		Acceptance rate of project quality $I_{53}$
		Pass rate of the project cost upon final accounting $I_{54}$
		Customer satisfaction $I_{55}$
		Capability of post-project evaluation $I_{56}$
		Capability of reusing project management experience $I_{57}$
		Emphasis on the impact on the ecological environment $I_{61}$
	Synergy process $I_{6}$	Cohesion capability of the project’s program management processes $I_{62}$
		Document integrity of project’s program management $I_{63}$
		Usage efficiency of project’s program management tool $I_{64}$
		Planning capabilities of project’s program management strategies $I_{65}$
		Level of multi-project management $I_{66}$

**Table 5:** Feature description of the environmental management maturity of CP.

Maturity Level of Environmental Management	Feature Description
Disordered level [0–1]	The implementation process of the CP is disorderly, only considers economic benefits and exhibits no awareness of environmental management
Simple level [1–2]	Consciousness of environmental management exists but only for the management of a single construction project without considering the overall concept of the CP.
Standard level [2–3]	The CP’s synergistic effect on the environment has been analyzed from the perspective of the whole, and the theoretical standards for environmental management of the CP have been constructed.
Improved level [3–4]	The implementation process of the CP is orderly, the environmental impact has been considered, the CP’s synergistic effect on the environment has been analyzed through qualitative and quantitative approaches, and countermeasures have been adopted at various stages of implementation.
Lean level [4–5]	The importance of environmental management has reached a certain degree, management tools have been continuously improved and optimized, and economic benefits have been sacrificed to achieve the purpose of environmental protection.

**Table 6:** Normalized judgment matrix and initial weight values of the criteria layer.

Index of the Criteria Layer	$I_{1}$	$I_{2}$	$I_{3}$	$I_{4}$	$I_{5}$	$I_{6}$	Initial Weight
$I_{1}$	0.0417	0.0156	0.0227	0.0204	0.0452	0.0637	0.0349
$I_{2}$	0.1250	0.0469	0.0227	0.0256	0.0271	0.0716	0.0532
$I_{3}$	0.1250	0.1406	0.0682	0.0341	0.0452	0.0818	0.0825
$I_{4}$	0.2083	0.1875	0.2045	0.1022	0.0679	0.0955	0.1443
$I_{5}$	0.1250	0.2344	0.2045	0.2044	0.1357	0.1146	0.1698
$I_{6}$	0.3750	0.3750	0.4773	0.6133	0.6787	0.5729	0.5154

## Appendix A. Judgment Matrix of the Index System

**Table A1:** Judgment matrix of the criteria layer index.

	$I_{1}$	$I_{2}$	$I_{3}$	$I_{4}$	$I_{5}$	$I_{6}$
$I_{1}$	1.000	0.333	0.333	0.200	0.333	0.111
$I_{2}$	3.000	1.000	0.333	0.250	0.200	0.125
$I_{3}$	3.000	3.000	1.000	0.333	0.333	0.143
$I_{4}$	5.000	4.000	3.000	1.000	0.500	0.167
$I_{5}$	3.000	5.000	3.000	2.000	1.000	0.200
$I_{6}$	9.000	8.000	7.000	6.000	5.000	1.000

**Table A2:** Judgment matrix of the initiating process index.

	$I_{11}$	$I_{12}$	$I_{13}$	$I_{14}$	$I_{15}$
$I_{11}$	1.00	0.33	0.20	0.17	0.13
$I_{12}$	3.00	1.00	0.33	0.20	0.17
$I_{13}$	5.00	3.00	1.00	0.33	0.20
$I_{14}$	6.00	5.00	3.00	1.00	0.33
$I_{15}$	8.00	6.00	5.00	3.00	1.00

**Table A3:** Judgment matrix of the planning process index.

	$I_{21}$	$I_{22}$	$I_{23}$	$I_{24}$	$I_{25}$
$I_{21}$	1.00	0.33	0.33	0.25	0.11
$I_{22}$	3.00	1.00	0.33	0.33	0.13
$I_{23}$	3.00	3.00	1.00	0.33	0.50
$I_{24}$	4.00	3.00	3.00	1.00	0.20
$I_{25}$	9.00	8.00	2.00	5.00	1.00

**Table A4:** Judgment matrix of the executing process index.

	$I_{31}$	$I_{32}$	$I_{33}$	$I_{34}$	$I_{35}$	$I_{36}$	$I_{37}$
$I_{31}$	1.00	0.50	0.50	0.33	0.25	0.17	0.13
$I_{32}$	2.00	1.00	0.20	0.25	0.20	0.20	0.11
$I_{33}$	2.00	5.00	1.00	0.50	0.33	0.14	0.25
$I_{34}$	3.00	4.00	2.00	1.00	0.33	0.20	0.20
$I_{35}$	4.00	5.00	3.00	3.00	1.00	0.33	0.33
$I_{36}$	6.00	5.00	7.00	5.00	3.00	1.00	0.20
$I_{37}$	8.00	9.00	4.00	5.00	3.00	5.00	1.00

**Table A5:** Judgment matrix of the controlling process index.

	$I_{41}$	$I_{42}$	$I_{43}$	$I_{44}$	$I_{45}$	$I_{46}$
$I_{41}$	1.00	0.25	0.25	0.20	0.14	0.13
$I_{42}$	4.00	1.00	0.33	0.25	0.50	0.20
$I_{43}$	4.00	3.00	1.00	0.33	0.33	0.25
$I_{44}$	5.00	4.00	3.00	1.00	0.50	0.25
$I_{45}$	7.00	2.00	3.00	2.00	1.00	0.50
$I_{46}$	8.00	5.00	4.00	4.00	2.00	1.00

**Table A6:** Judgment matrix of the closing process index.

	$I_{51}$	$I_{52}$	$I_{53}$	$I_{54}$	$I_{55}$	$I_{56}$	$I_{57}$
$I_{51}$	1.00	0.20	0.50	0.25	0.14	0.17	0.11
$I_{52}$	5.00	1.00	0.33	0.33	0.25	0.20	0.17
$I_{53}$	2.00	3.00	1.00	0.50	0.33	0.33	0.25
$I_{54}$	4.00	3.00	2.00	1.00	0.33	0.17	0.33
$I_{55}$	7.00	4.00	3.00	3.00	1.00	0.20	0.50
$I_{56}$	6.00	5.00	3.00	6.00	5.00	1.00	0.50
$I_{57}$	9.00	6.00	4.00	3.00	2.00	2.00	1.00

**Table A7:** Judgment matrix of the synergy process index.

	$I_{61}$	$I_{62}$	$I_{63}$	$I_{64}$	$I_{65}$	$I_{66}$
$I_{61}$	1.00	0.17	0.20	0.17	0.17	0.13
$I_{62}$	6.00	1.00	0.50	0.33	0.17	0.17
$I_{63}$	5.00	2.00	1.00	0.50	0.25	0.25
$I_{64}$	6.00	3.00	2.00	1.00	0.25	0.20
$I_{65}$	6.00	6.00	4.00	4.00	1.00	0.33
$I_{66}$	8.00	6.00	4.00	5.00	3.00	1.00

## Appendix B. Importance Score Set of Each Expert to Others

**Table A8:** Importance score set of each expert to others.

		$I_{1}$	$I_{2}$	$I_{3}$	$I_{4}$	$I_{5}$	$I_{6}$
Scores of 1st expert	1st expert	5	5	5	5	5	5
	2nd expert	4	5	4	3	4	5
	3rd expert	4	3	2	1	5	3
	4th expert	3	3	4	5	4	2
	5th expert	2	5	5	3	3	5
Scores of 2nd expert	1st expert	3	5	4	3	3	4
	2nd expert	5	5	5	5	5	5
	3rd expert	4	4	3	3	1	2
	4th expert	4	2	5	3	3	2
	5th expert	3	3	4	4	3	4
Scores of 3rd expert	1st expert	4	3	5	3	3	3
	2nd expert	3	3	3	4	2	4
	3rd expert	5	5	5	5	5	5
	4th expert	3	1	4	3	2	4
	5th expert	4	4	3	3	4	2
Scores of 4th expert	1st expert	4	4	4	2	3	4
	2nd expert	3	4	2	4	3	3
	3rd expert	5	3	4	3	3	3
	4th expert	5	5	5	5	5	5
	5th expert	4	3	4	3	4	1
Scores of 5th expert	1st expert	4	3	3	4	4	4
	2nd expert	4	5	4	2	4	5
	3rd expert	4	3	4	2	2	2
	4th expert	3	2	4	4	3	2
	5th expert	5	5	5	5	5	5

## Appendix C. Status Levels Scored by Five Experts

**Table A9:** Scores of the status level of the criteria layer index.

		$I_{1}$	$I_{2}$	$I_{3}$	$I_{4}$	$I_{5}$	$I_{6}$
Scores of 1st expert	$CP_{1}$	$I_{2}$	$I_{3}$	$I_{4}$	$I_{5}$	$I_{6}$	4
	$CP_{2}$	3	3	4	5	2	3
	$CP_{3}$	4	4	3	3	4	5
	$CP_{4}$	4	3	4	2	2	4
Scores of 2nd expert	$CP_{1}$	4	4	4	5	4	5
	$CP_{2}$	5	3	3	3	3	4
	$CP_{3}$	5	5	4	3	4	3
	$CP_{4}$	3	2	3	3	4	3
Scores of 3rd expert	$CP_{1}$	5	4	2	4	3	5
	$CP_{2}$	4	3	3	2	4	5
	$CP_{3}$	4	3	2	3	2	5
	$CP_{4}$	5	5	4	1	4	3
Scores of 4th expert	$CP_{1}$	4	4	3	4	3	4
	$CP_{2}$	3	4	3	3	3	4
	$CP_{3}$	4	3	3	2	3	4
	$CP_{4}$	3	4	3	2	4	5
Scores of 5th expert	$CP_{1}$	4	5	5	4	3	4
	$CP_{2}$	3	2	4	4	3	4
	$CP_{3}$	5	3	3	1	5	4
	$CP_{4}$	4	4	4	3	5	5

**Table A10:** Scores of the status level of the initiating process index.

		$I_{11}$	$I_{12}$	$I_{13}$	$I_{14}$	$I_{15}$
Scores of 1st expert	$CP_{1}$	3	3	3	3	4
	$CP_{2}$	2	4	5	3	3
	$CP_{3}$	4	3	3	5	4
	$CP_{4}$	4	4	4	3	2
Scores of 2nd expert	$CP_{1}$	5	5	4	3	5
	$CP_{2}$	2	3	3	4	4
	$CP_{3}$	3	5	4	3	4
	$CP_{4}$	4	4	4	2	3
Scores of 3rd expert	$CP_{1}$	4	4	3	5	4
	$CP_{2}$	3	5	4	4	3
	$CP_{3}$	3	4	3	5	4
	$CP_{4}$	2	4	4	3	4
Scores of 4th expert	$CP_{1}$	3	5	3	4	5
	$CP_{2}$	2	4	4	4	4
	$CP_{3}$	3	5	3	3	4
	$CP_{4}$	3	3	4	2	4
Scores of 5th expert	$CP_{1}$	2	4	4	2	3
	$CP_{2}$	3	5	2	3	5
	$CP_{3}$	4	4	3	3	4
	$CP_{4}$	4	4	3	2	3

**Table A11:** Scores of the status level of the planning process index.

		$I_{21}$	$I_{22}$	$I_{23}$	$I_{24}$	$I_{25}$
Scores of 1st expert	$CP_{1}$	3	3	5	4	2
	$CP_{2}$	3	4	5	3	4
	$CP_{3}$	5	4	2	4	4
	$CP_{4}$	4	5	3	3	4
Scores of 2nd expert	$CP_{1}$	3	5	4	2	3
	$CP_{2}$	3	2	4	2	3
	$CP_{3}$	2	3	5	4	2
	$CP_{4}$	4	2	4	5	3
Scores of 3rd expert	$CP_{1}$	3	3	4	1	3
	$CP_{2}$	4	4	3	3	5
	$CP_{3}$	2	4	4	3	3
	$CP_{4}$	3	3	4	1	3
Scores of 4th expert	$CP_{1}$	1	3	4	1	5
	$CP_{2}$	4	3	3	1	3
	$CP_{3}$	4	2	3	4	3
	$CP_{4}$	3	2	4	5	3
Scores of 5th expert	$CP_{1}$	4	3	2	3	4
	$CP_{2}$	4	3	5	5	5
	$CP_{3}$	4	3	3	3	3
	$CP_{4}$	2	5	1	2	4

**Table A12:** Scores of the status level of the executing process index.

		$I_{31}$	$I_{32}$	$I_{33}$	$I_{34}$	$I_{35}$	$I_{36}$	$I_{37}$
Scores of 1st expert	$CP_{1}$	4	2	4	4	3	5	5
	$CP_{2}$	5	4	3	4	4	3	3
	$CP_{3}$	3	4	3	2	3	2	5
	$CP_{4}$	4	4	4	3	4	3	3
Scores of 2nd expert	$CP_{1}$	5	3	3	4	3	3	4
	$CP_{2}$	3	5	3	5	3	3	4
	$CP_{3}$	4	4	4	3	3	5	3
	$CP_{4}$	4	4	3	3	4	3	3
Scores of 3rd expert	$CP_{1}$	5	4	3	3	5	3	4
	$CP_{2}$	4	5	5	4	3	4	3
	$CP_{3}$	3	2	3	2	3	5	5
	$CP_{4}$	5	4	4	3	3	3	5
Scores of 4th expert	$CP_{1}$	3	5	3	3	3	3	5
	$CP_{2}$	4	3	4	4	2	4	3
	$CP_{3}$	2	4	3	2	3	2	5
	$CP_{4}$	3	4	4	4	3	3	5
Scores of 5th expert	$CP_{1}$	3	5	3	2	3	4	5
	$CP_{2}$	3	2	3	3	5	3	3
	$CP_{3}$	5	5	4	3	4	3	4
	$CP_{4}$.	3	4	4	4	3	4	5

**Table A13:** Scores of the status level of the controlling process index.

		*I*41	*I*42	*I*43	*I*44	*I*45	*I*46
Scores of 1st expert	$CP_{1}$	4	3	3	1	4	5
	$CP_{2}$	2	5	4	3	4	5
	$CP_{3}$	4	3	4	3	4	4
	$CP_{4}$	4	5	2	4	5	3
Scores of 2nd expert	$CP_{1}$	3	4	2	4	3	4
	$CP_{2}$	3	4	3	4	4	3
	$CP_{3}$	4	4	4	3	4	3
	$CP_{4}$	4	3	4	5	4	3
Scores of 3rd expert	$CP_{1}$	3	4	2	3	3	5
	$CP_{2}$	5	3	5	4	3	4
	$CP_{3}$	3	4	4	3	2	3
	$CP_{4}$	3	4	3	3	4	4
Scores of 4th expert	$CP_{1}$	4	4	3	4	4	5
	$CP_{2}$	3	5	3	4	34	4
	$CP_{3}$	3	4	5	5	4	3
	$CP_{4}$	4	2	5	4	1	2
Scores of 5th expert	$CP_{1}$	4	5	3	3	4	2
	$CP_{2}$	2	4	4	3	1	3
	$CP_{3}$	2	2	2	2	2	3
	$CP_{4}$	2	1	3	4	4	1

**Table A14:** Scores of the status level of the closing process index.

		$I_{51}$	$I_{52}$	$I_{53}$	$I_{54}$	$I_{55}$	$I_{56}$	$I_{57}$
Scores of 1st expert	$CP_{1}$	3	3	4	3	4	2	5
	$CP_{2}$	3	3	5	3	4	4	5
	$CP_{3}$	3	5	4	4	4	4	4
	$CP_{4}$	4	1	1	2	3	3	3
Scores of 2nd expert	$CP_{1}$	3	2	2	2	4	3	3
	$CP_{2}$	4	1	3	3	3	4	3
	$CP_{3}$	2	3	3	4	5	4	4
	$CP_{4}$	4	2	2	3	3	3	4
Scores of 3rd expert	$CP_{1}$	5	1	2	2	2	3	4
	$CP_{2}$	4	4	4	3	3	3	3
	$CP_{3}$	3	5	3	4	5	4	4
	$CP_{4}$	5	2	2	2	3	3	3
Scores of 4th expert	$CP_{1}$	3	3	4	3	2	3	3
	$CP_{2}$	4	4	3	3	5	4	4
	$CP_{3}$	3	4	5	4	4	5	4
	$CP_{4}$	3	3	3	2	2	4	1
Scores of 5th expert	$CP_{1}$	5	4	4	4	5	4	4
	$CP_{2}$	4	4	4	3	2	3	3
	$CP_{3}$	4	3	3	4	5	4	4
	$CP_{4}$	3	2	4	4	2	2	5

**Table A15:** Scores of the status level of the synergy process index.

		$I_{61}$	$I_{62}$	$I_{63}$	$I_{64}$	$I_{65}$	$I_{66}$
Scores of 1st expert	$CP_{1}$	3	3	2	4	4	4
	$CP_{2}$	3	4	2	3	3	3
	$CP_{3}$	3	4	3	4	4	4
	$CP_{4}$	3	4	3	3	4	2
Scores of 2nd expert	$CP_{1}$	4	3	4	3	4	5
	$CP_{2}$	3	3	3	3	4	4
	$CP_{3}$	3	5	3	3	5	3
	$CP_{4}$	5	4	3	4	2	4
Scores of 3rd expert	$CP_{1}$	5	3	5	4	3	5
	$CP_{2}$	4	4	3	4	4	4
	$CP_{3}$	3	4	3	3	3	3
	$CP_{4}$	3	3	1	2	2	5
Scores of 4th expert	$CP_{1}$	4	4	1	3	3	5
	$CP_{2}$	4	3	3	3	5	3
	$CP_{3}$	2	1	2	4	4	4
	$CP_{4}$	3	2	4	2	2	2
Scores of 5th expert	$CP_{1}$	3	5	3	2	5	3
	$CP_{2}$	3	3	3	4	2	4
	$CP_{3}$	4	2	3	3	4	4
	$CP_{4}$	5	3	1	2	5	3

## Appendix D. Environmental Management Maturity Level Scored by Five Experts

**Table A16:** Scores of environmental management maturity in the criteria layer index.

	$I_{1}$	$I_{2}$	$I_{3}$	$I_{4}$	$I_{5}$	$I_{6}$
1st expert	1.5	2.9	3.3	1.9	4.1	0.8
2nd expert	1.8	3.8	3.2	2.4	4.8	1.1
3rd expert	1.9	3.1	2.6	2.1	3.6	1.8
4th expert	2.4	3.4	3.8	1.9	3.5	1.6
5th expert	2.8	3.3	3.1	3.2	3.1	1.4

**Table A17:** Scores of environmental management maturity in the initiating process index.

	$I_{11}$	$I_{12}$	$I_{13}$	$I_{14}$	$I_{15}$
1st expert	1.2	1.4	2.1	2.4	0.9
2nd expert	1.5	0.9	1.3	1.4	1.8
3rd expert	1.7	1.7	1.8	2.1	1.9
4th expert	1.9	2.1	2.2	2.3	2.6
5th expert	2.9	3.2	2.6	3	2.4

**Table A18:** Scores of environmental management maturity in the planning process index.

	$I_{21}$	$I_{22}$	$I_{23}$	$I_{24}$	$I_{25}$
1st expert	2.7	2.9	3.2	3.1	3.4
2nd expert	3.6	3.9	4.0	4.1	3.9
3rd expert	2.8	2.6	3.2	2.8	2.7
4th expert	3.3	3.4	3.6	3.8	3.6
5th expert	3.6	3.4	2.4	2.6	3.3

**Table A19:** Scores of environmental management maturity in the executing process index.

	$I_{31}$	$I_{32}$	$I_{33}$	$I_{34}$	$I_{35}$	$I_{36}$	$I_{37}$
1st expert	2.8	2.7	3.6	3.5	3.8	2.2	4.1
2nd expert	3.1	2.9	2.9	3.1	2.6	3.5	3.4
3rd expert	1.9	2.2	1.5	2.6	3.6	3.8	3.2
4th expert	3.8	3.7	3.8	3.9	3.8	3.6	3.7
5th expert	3.3	3.4	3.3	2.9	2.8	2.8	3.5

**Table A20:** Scores of environmental management maturity in the controlling process index.

	$I_{41}$	$I_{42}$	$I_{43}$	$I_{44}$	$I_{45}$	$I_{46}$
1st expert	2.1	2.3	3.1	0.9	1.3	3.8
2nd expert	1.9	2.8	2.7	2.6	2.8	2.8
3rd expert	2.3	2.4	2.8	3.1	2.7	5.6
4th expert	2.4	0.8	2.8	2.1	1.9	1.9
5th expert	3.3	3.4	2.9	2.8	3.5	3.6

**Table A21:** Scores of environmental management maturity in the closing process index.

	$I_{51}$	$I_{52}$	$I_{53}$	$I_{54}$	$I_{55}$	$I_{56}$	$I_{57}$
1st expert	3.8	3.9	4.3	4.3	4.6	4.3	4.2
2nd expert	4.5	3.9	3.8	3.9	4.3	4.5	4.6
3rd expert	3.7	3.9	3.4	3.3	3.5	4.1	3.9
4th expert	3.7	3.9	3.5	3.6	3.6	3.7	3.6
5th expert	2.9	3.3	3.2	3.4	2.9	3.1	3.3

**Table A22:** Scores of environmental management maturity in the synergy process index.

	$I_{61}$	$I_{62}$	$I_{63}$	$I_{64}$	$I_{65}$	$I_{66}$
1st expert	1.1	1.2	0.6	0.8	0.9	1.3
2nd expert	1.7	1.8	1.4	1.3	1.5	0.9
3rd expert	1.9	2.1	2.3	1.9	1.7	2.1
4th expert	2.7	2.4	2.5	0.8	2.2	2.1
5th expert	1.6	1.8	1.9	2.1	2.1	2.4
